# Studies on the Feeding Habits of *Lutzomyia (Lutzomyia) longipalpis* (Lutz & Neiva, 1912) (Diptera: Psychodidae: Phlebotominae) Populations from Endemic Areas of American Visceral Leishmaniasis in Northeastern Brazil

**DOI:** 10.1155/2012/858657

**Published:** 2012-01-18

**Authors:** Margarete Martins dos Santos Afonso, Rosemere Duarte, José Carlos Miranda, Lindenbergh Caranha, Elizabeth Ferreira Rangel

**Affiliations:** ^1^Laboratório de Transmissores de Leishmanioses, Laboratório de Referência em Vigilância Entomológica, Taxonomia e Ecologia de Vetores das Leishmanioses do Instituto Oswaldo Cruz, FIOCRUZ, Avenida Brasil, 4365, 21040-360 Manguinhos, RJ, Brazil; ^2^Laboratório de Pesquisa e Serviços em Saúde Pública, Departamento de Ciências Biológicas, Escola Nacional de Saúde Pública Sérgio Arouca, FIOCRUZ, Rua Leopoldo Bulhões, 1480, 21041-210 Manguinhos, RJ, Brazil; ^3^Laboratório de Imunoparasitologia, Centro de Pesquisas Gonçalo Moniz, FIOCRUZ, Rua Valdemar Falcão, 121, 40296-710 Salvador, BA, Brazil; ^4^Laboratório de Vetores, Reservatórios e Animais Peçonhentos Dr. Thomaz Aragão SESA/CE, Rua dos Tabajaras, 268, Praia de Iracema, Fortaleza, 60060-510 CE, Brazil

## Abstract

The aim of this study was to identify potential blood feeding sources of *L. (L.) longipalpis* specimens from populations in Northeastern Brazil, endemic areas of American Visceral Leishmaniasis (AVL) and its correlation with the transmission of *L. (L.) i. chagasi*. The ELISA technique was applied using bird, dog, goat, opossum, equine, feline, human, sheep, and rodent antisera to analyze 609 females, resulting in an overall positivity of 60%. In all municipalities, females showed higher positivity for bird followed by dog antiserum and sand fly specimens were also positive for equine, feline, human, sheep, goat, opossum, and rodent antisera. The finding for 17 combinations of two or three types of blood in some females corroborates the opportunistic habit of this sand fly species. The results demonstrating the association between *L. (L.) longipalpis* and opossum suggest the need for further evaluation of the real role of this synanthropic mammal in the eco-epidemiology of AVL.

## 1. Introduction

American visceral leishmaniasis (AVL) is a serious public health problem in Brazil and presents a new epidemiological profile associated with domestic environments and, in this context, *Lutzomyia (Lutzomyia) longipalpis* is important considering its capacity to adapt to a wide range of impacted habitats, in addition to its sylvatic origin [1–3]. The enzyme immunoassay (ELISA) has been used to identify the feeding habits of *L. (L.) longipalpis* [4–6].

In light of this, studies related to feeding habits of sand fly vector *L. (L.) longipalpis* could contribute to a better understanding of eco-epidemiology of AVL, discussing its close association with *Leishmania (Leishmania) infantum chagasi* reservoirs.

Currently, Northeastern Brazil accounts for about 47% of human cases for AVL exhibiting both epidemiological profiles, rural and urban, with highest incidences of the disease according to the Brazilian National Leishmaniases Program (NLP) [1]. The aim of this study was to identify potential blood meal sources for *L. (L.) longipalpis *from some Northeastern Brazil endemic municipalities.

## 2. Materials and Methods

### 2.1. Sand Fly Precedence

Sand flies were collected from the municipalities of Jequié (State of Bahia, BA), Sobral and Massapê (State of Ceará, CE), and Teresina (State of Piauí, PI). These municipalities were selected based on their levels and profiles of AVL transmission according to NLP: Teresina, urban and intense transmission; Sobral, rural and intense transmission; Jequié, rural and moderate transmission, and Massapê, rural and sporadic transmission [1]. Sand flies were collected from March 2006 to September 2007 during months of high frequency of sand flies.

### 2.2. Sand Fly Sampling

Sand flies were collected using unbaited modified CDC light traps [7], HP model [8], between 6:00 PM and 6:00 AM For each location (municipality), the same number of site collections was selected (four houses with human cases of AVL and with environmental characteristics adequate for breeding sand flies). Collections were done in the peridomicile; two traps were randomly placed at a minimum distance of 50 meters from the residence and from each other. The collected sand flies were screened in a cold chamber (cryolyzer), and females were stored in plastic microtubes and placed in a −20°C freezer for future analysis.

### 2.3. Bloodmeal Analysis

*L. (L.) longipalpis* engorged females or those with some residual blood meal were tested by ELISA, following the method of Burkot et al. [9] modified by Duarte [10] using bird, dog, goat, opossum, equine, feline, human, sheep, and rodent antisera; male specimens of *L. (L.) longipalpis *were used as a control; they were treated in the same manner as the overfed female samples. The males for the negative control were chosen because they do not feed on blood and were used to pinpoint false positives in the samples of female sand flies. The choice of antisera was based on the most common animals at collection sites. The *L. (L.) longipalpis *specimens were identified according to diagnostic morphological features (cibarium and spermatheca) [11].

To the analysis, the abdomen of the frozen female specimens, as well as the controls (males), were macerated in PBS (pH 7.2, 0.001 M) and kept at −20°C until processing. Samples were diluted 1 : 20 in carbonate bicarbonate buffer (pH 9.6; 0.05 M, Sigma) and applied to 96-well polystyrene microplate (NuncC, 442404, maxisorp, Dermark). After incubation (at 37°C for 2 h), plates were washed in PBS/Tween 20–0.05% (Sigma Chemicals Co-St. Louis, USA). The next steps involved adding antiserum (PBS/Tween 20 plus 1% skim milk—Molico-Nestlé, Brazil) into the wells and incubating the microplate at 37°C for 30 min (goat anti-rabbit serum peroxidase conjugate—Sigma Chemical USA). Should be washed, the diluted conjugate, at 1 : 20,000 was added, and after another incubation and wash, the developing buffer (citrate/phosphate pH 5.0–0.05 M hydrogen peroxide (H_2_O_2_) 30 vol. Merck Diagnóstica-RJ, Brasil and O-Phenylenediamine—OPD, Sigma Immunochemicals Co. USA) was applied. The reaction was stopped after 15 min by adding 50 *μ*L of sulfuric acid solution 1 N and read on an ELISA plate reader (WI, USA) using 490 nm operational and 630 nm reference filters.

In every plate, positive controls were used consisting of homologous sera diluted in carbonate bicarbonate buffer (pH 9.6, 0.05 M, Sigma) 200 times. For validation, amounts over 1.0 were expected. In order to estimate the positivity of the samples, a calculation was made based on the average of the absorbance obtained from the reactions observed in heterologous serums plus two standard deviations (cutoff point). This procedure was adopted to exclude the results of possible crossover reactions and to increase the specificity of the assay.

The antisera used were obtained from the Immunodiagnostics Laboratory, Department of Biological Science, *Escola Nacional de Saúde Pública Sérgio Arouca*, FIOCRUZ; because there are no antibodies to all food sources, we used anti-total protein, which can increase the sensitivity of the method, since the insect feeds on blood. The assay sensibility is estimated (96%) in the Standard Operational Procedure described in the Laboratory where the analyses were performed and described in the reference Duarte [10].

## 3. Results

A total of 609 sand fly females were analyzed and a general reactivity index of 60% was obtained; specimens from Massapê displayed the greatest diversity in feeding sources, 19 antisera or combinations of antisera unlike the population from Jequié, which was reactive to only six. The percentages of positivity to the antisera tested in four populations of *L. (L.) longipalpis* are presented in Table 1.

Bird, dog, and equine antisera were positive for all *L. (L.) longipalpis* populations, with highest positivity observed in blood of birds (Jequié, 36.0%, Teresina, 67.6%, Sobral, 29.9%, Massapê, 51.0%) followed by blood of dogs (Jequié, 16.0%, Teresina 9.5%, Sobral 14.3%, Massapê 2.3%). Equine antisera were reactive in all populations (Jequié, 16.0%, Teresina 2.7%, Sobral 15.6%, Massapê 8.5%). Blood from opossum was detected in *L. (L.) longipalpis *females from three populations (Jequié, 8.0%, Sobral, 2.6%, and Massapê, 2.3%), and blood of sheep has also been identified in three populations (Teresina 2.7%, Sobral 9.1%, and Massapê 10.8%). Human antiserum was reactive in *L. (L.) longipalpis *females from Jequié (20%) and Massapê (0.8%). Feline and goat antisera showed positivity for females from Sobral and Massapê, respectively. And rodent antisera were reactive only in Massapê species.

Females that had fed on more than one blood meal source for all *L. (L.) longipalpis *populations were detected; especially those from Sobral and Massapê, specimens were found that had fed on at least three distinct sources. From the 17 blood combinations detected, dog + bird, dog + sheep, dog + equine, dog + opossum, sheep + feline, and goat + sheep + equine were the most frequent.

## 4. Discussion

Previous reports based on field studies have suggested that *L. (L.) longipalpis *has a varied diet, feeding on a wide range of animals, including dogs, pigs, horses, cattle, and chickens [3, 12–14]. Along with favorable environmental conditions, it has been suggested that population growth of this sand fly vector is determined by the abundance of food sources, which facilitates its adaptation to human dwellings, especially in rural areas.

The data from the present study show that specimens from the four analyzed populations fed mainly on birds, based on the significant percentage of positivity compared to other feeding sources. The strong attraction to birds has already been observed in many other studies in Brazilian States such as Maranhão (MA) [15–17] and Mato Grosso do Sul (MS) [5]. It is known that when chicken coops are in a peridomicile, they attract sand flies and can act as breeding sites. This proximity increases contact between vectors and humans, suggesting that chicken coops play an important epidemiological role [18].^.^Although chickens may be refractory to *Leishmania *infection; they serve as a feeding source for sand flies and attract potential reservoirs of *L. (L.) i. chagasi*, allowing the establishment and maintenance of AVL transmission in rural areas [19]. In Argentina, a study on the spatial distribution of *L. (L.) longipalpis* found a positive association between the vector and the presence of chicken coops and could contribute to the design of control strategies, defining priority areas for prevention and control [20].

The positivity to dog blood was confirmed for the *L. (L.) longipalpis *populations analyzed. Since the pioneering studies on epidemiology of AVL in Brazil (Sobral/CE), the attraction to dogs was evident [12]. In most AVL transmission areas, there is epidemiological evidence implicating dogs as domestic reservoirs [3]. Studies have been suggesting that dogs are an important blood source for *L. (L.) longipalpis *in Teresina (PI) and Araçatuba (SP), both areas for high incidence of AVL [21, 22]. However, studies conducted in Campo Grande (MS), an area with high prevalence of AVL human cases and where the presence of dogs was observed in all domestic sites, showed a low reactivity for dog blood [5]. In this way, sand fly populations may present different feeding preferences in different ecotypes.

A low positive reaction to feline antiserum was only found in *L. (L.) longipalpis *females from Sobral and Massapê. In fact, there is no evidence for a role of felines in the AVL epidemiology, although recently the experimental infection of *L. (L.) longipalpis* by *L. infantum* (= *L. infantum chagasi*) was described after xenodiagnosis on a naturally infected cat from an endemic area in Belo Horizonte (MG) [23].

In the present study, even with low indices of positivity, opossum blood in specimens from three populations was detected: Sobral, Massapê, and Jequié. This is the first report of an association between* L. (L.) longipalpis *and opossums in Brazil. In Colombia, a low reactivity for opossums was also observed in *L. (L.) longipalpis *[24]. These data should be analyzed carefully since the opossum (*Didelphis albiventris*) has been suggested as a possible secondary reservoir host for *L. (L.) i. chagasi*; based on studies conducted in Jacobina/BA, only 2 of 84 animals were positive, a result that for some authors has no significant epidemiological importance [25, 26]. Subsequently, other studies have reported natural infections of *Didelphis marsupialis *by *Leishmania *spp., possibly *L. (L.) i. chagasi*, arguing the role of these mammals as potential AVL reservoirs [27, 28]. In light of the evidence for natural infection in opossums and the finding that these animals are feeding sources for *L. (L.) longipalpis, *the possibility that this synanthropic mammal participates in the transmission cycle of AVL in some regions should be considered, especially those that have been undergoing environmental alterations, which facilitates contact of these animals with human habitation.

The anthropophily of *L. (L.) longipalpis *has been observed in fieldworks [1–3, 12, 19], which is one essential criteria in implicating sand fly species as vector. Otherwise, studies evaluating the blood meal of this sand fly conducted in Marajó Island (State of Pará) and in Campo Grande (MS) demonstrated its high anthropophily [4, 5]. Only two *L. (L.) longipalpis* populations (Jequié and Massapê) in this study contained human blood in low percentage. Similar results obtained in Araçatuba suggested low anthropophily of *L. (L.) longipalpis *[22], which was also observed in studies conducted in Colombia [24]. The abundance of feeding sources (birds and dogs) surrounding houses should be considered, as observed at the collection sites in Teresina and Sobral, it can be a determining factor in the choice of the insect's feeding source, corroborating the opportunistic behavior of this sand fly.

The finding of more than one blood source in some females, mainly in Massapê (up to three), is a strong evidence of the eclectic diet of *L. (L.) longipalpis*. This behavior is common in sand flies, which is to “try out” different hosts until completing their blood meal. This is, undoubtedly, an important aspect in leishmaniases transmission.

The adaptation of *L. (L.) longipalpis *to different habitats, even its urbanization, remains a great challenge for AVL control. Clearly, the results of this study indicate the eclecticism of *L. (L.) longipalpis *with regard to blood feeding. In light of entomological surveillance of leishmaniases, information about the biology of the vector, and especially its interaction with reservoir hosts in association with environmental factors, can indicate changes in the transmission profile of AVL and the process of geographical expansion [29]. In this context, the ability to feed frequently on domestic animals such as birds (chickens), dogs and even synanthropic mammals (opossums) is an important attribute that allows *L. (L.) longipalpis *to maintain AVL transmission in rural environments and the expansion of the disease to urban areas, contributing to the maintenance of two transmission profiles currently found in Brazil.

##  Funding

This paper was supported by Instituto Oswaldo Cruz and Escola Nacional de Saúde Pública Sergio Arouca-FIOCRUZ; Conselho Nacional de Desewnvolvimento Científico e Tecnológico, DECIT-CNPq/2006 (# 410503/2006-1).

## Figures and Tables

**Table 1:** Percentage of positive females of *Lutzomyia (Lutzomyia) longipalpis*. analyzed by ELISA collected in the Municipality of Jequié (BA), Teresina (PI), Sobral (CE) and Massapê (CE), Northeast Brazil.

Municipality				
Antisera reactivity %	Jequié (BA)	Teresina (PI)	Sobral (CE)	Massapê (CE)
Bird	36.0	67.6	29.9	51.0
Dog	16.0	9.5	14.3	2.3
Goat	—	—	—	—
Opossum	8.0	—	2.6	2.3
Equine	16.0	2.7	15.6	8.5
Feline	—	—	1.3	—
Human	20.0	—	—	0.8
Sheep	—	2.7	9.1	10.8
Rodent	—	—	—	0.8
Dog + Human	4.0	—	—	—
Dog + Bird	—	4.1	—	2.3
Dog + Sheep	—	8.1	3.9	4.6
Dog + Equine	—	—	7.8	3.9
Equine + Sheep	—	—	2.6	—
Goat + Equine	—	—	2.6	—
Dog + Opossum	—	—	1.3	3.1
Sheep + Feline	—	—	1.3	0.8
Bird + Equine	—	—	1.3	—
Bird + Opossum	—	—	—	1.5
Bird + Sheep	—	—	—	0.8
Human + Sheep	—	—	—	0.8
Dog + Human + Equine	—	—	—	1.5
Bird + Dog + Equine	—	—	—	0.8
Bird + Opossum + Dog	—	—	—	0.8
Goat + Sheep + Equine	—	—	5.2	2.3
Dog + Sheep + Bird	—	5.4	—	—
